# CircRNAs: A Promising Star for Treatment and Prognosis in Oral Squamous Cell Carcinoma

**DOI:** 10.3390/ijms241814194

**Published:** 2023-09-17

**Authors:** Mengyi Zhu, Daoyang Chen, Chuangdong Ruan, Penghui Yang, Jinrong Zhu, Rongxin Zhang, Yan Li

**Affiliations:** Department of Biotechnology, School of Life Sciences and Biopharmaceutics, Guangdong Pharmaceutical University, Guangzhou 510006, China; zhumengyihi@163.com (M.Z.); staog5174@gmail.com (D.C.); dong123123122023@163.com (C.R.); zhujinrong@gdpu.edu.cn (J.Z.)

**Keywords:** circRNA, OSCC, biomarker, miRNA sponge

## Abstract

CircRNAs are a class of endogenous long non-coding RNAs with a single-stranded circular structure. Most circRNAs are relatively stable, highly conserved, and specifically expressed in tissue during the cell and developmental stages. Many circRNAs have been discovered in OSCC. OSCC is one of the most severe and frequent forms of head and neck cancer today, with a poor prognosis and low overall survival rate. Due to its prevalence, OSCC is a global health concern, characterized by genetic and epigenomic changes. However, the mechanism remains vague. With the advancement of biotechnology, a large number of circRNAs have been discovered in mammalian cells. CircRNAs are dysregulated in OSCC tissues and thus associated with the clinicopathological characteristics and prognosis of OSCC patients. Research studies have demonstrated that circRNAs can serve as biomarkers for OSCC diagnosis and treatment. Here, we summarized the properties, functions, and biogenesis of circRNAs, focusing on the progress of current research on circRNAs in OSCC.

## 1. Introduction

Oral squamous cell carcinoma (OSCC) is a kind of head and neck cancer (HNC) with a high mortality rate and a survival rate of only about 50% [1]. OSCC, a diverse category of malignancies, originates in the oral cavity’s mucosal lining [2]. OSCC is recognized as being caused by a variety of factors, including heredity and risky lifestyle behaviors like alcohol use, tobacco use, betel quid chewing [3], and HPV [4]. According to the severity of the cancer and the patient’s co-morbid conditions, OSCC can be treated with either surgical treatment, chemotherapy, radiotherapy, immunotherapy, photodynamic therapy (PDT), or a combination of these modalities [5]. However, the survival of OSCC patients has not improved significantly in recent years, with locoregional recurrence being the leading cause of therapeutic failure [6,7]. Furthermore, patients with OSCC are generally diagnosed at an advanced stage, thus posing a greater challenge for treatment. Therefore, finding new and precise diagnostic and therapeutic approaches to OSCC is a critical issue.

Circular RNA (CircRNA), a single-stranded closed-loop RNA that lacks terminal 5′ caps and 3′ poly (A) tails, is more stable than linear RNA [8]. CircRNA was first discovered in plant-infected viroids [9] and was later observed in eukaryotes, although it was not produced via the back-splicing mechanism [10]. Increasing numbers of differentially expressed circRNAs have been identified as a result of improvements in high-throughput sequencing techniques and bioinformatics. Over the years, a variety of the biological functions of circRNAs have been disclosed. An increasing number of studies have suggested that circRNAs are involved in a variety of diseases and may serve as biomarkers for cancer diagnostic and therapeutic targets [11]. Notably, circRNAs play essential roles in cell proliferation, apoptosis, migration, and invasion, thus regulating the progression of OSCC. Therefore, exploring OSCC-related circRNAs may open up new avenues for the early detection, prognosis, and effective therapy of OSCC. To gain a detailed understanding of the impact of circRNAs on OSCC, we developed a novel search strategy: (CircularRNA OR circRNA OR “CircularRNA” OR circ*) AND (“Oral square cell carcinoma” OR OSCC). Due to the different ways in which different databases can be used, their formats may vary slightly. In addition, we limited the time period of the publications to 2017–2023. A total of 339 articles were found (163 from PubMed, 160 from Web of Science, and 8 from Science Direct). After removing duplicates, 167 articles met the inclusion criteria. Zotero software (version 6.0) was used to manage the references. From these studies, we summarized the dysregulation, roles, and clinical consequences of circRNAs in OSCC.

## 2. Characteristics, Biogenesis and Functions of CircRNAs

### 2.1. Characteristics

Through in-depth studies of circRNAs, scientists have obtained a fundamental understanding of the characteristics of circRNAs, including their diversity, expression, stability, and conservation.

- Diversity: from fruit flies to humans, circRNAs have been discovered in a diverse range of metazoans, cell types, and species. Additionally, circRNAs have been detected in plants and other organisms;
- Highly abundant expression: circRNAs are more abundantly distributed in human cells than their equivalent linear mRNAs, according to Salzaman [12];
- Stability: due to their distinctive topology, circRNAs, lacking 5′ caps and 3′ tails in comparison with other non-coding RNAs, are more stable than linear RNAs [13]. In addition, owing to their resistance to RNA exonucleases or RNase R [14], circRNAs exhibit a half-life over 48 h in most species [13];
- Conservation: the majority of circRNAs appear to be highly conservative as a result of splicing. Most circRNAs are highly conservative in various species, with only a few exceptions being unconservative [15].

### 2.2. Biogenesis

**Figure 1:**
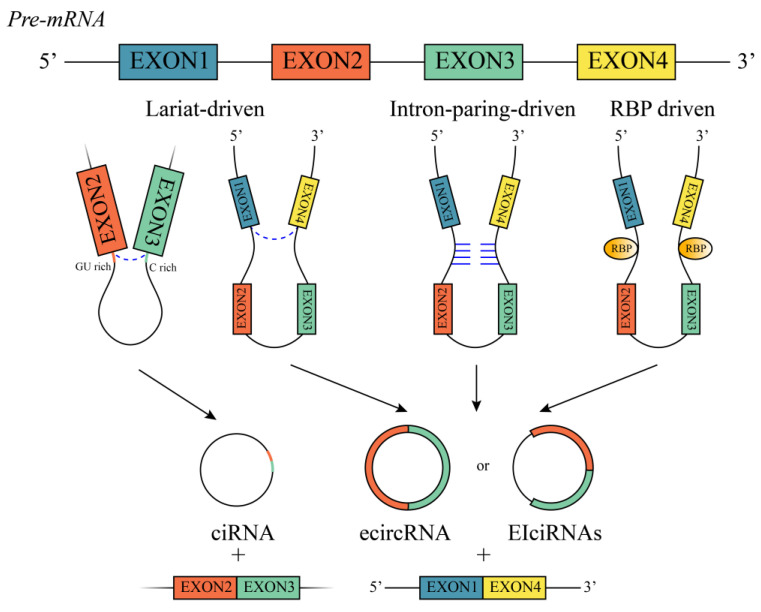
Biogenesis of circRNAs. Schematic showing three commonly recognized circRNA formations: lariat-driven circularization, intron-pair-driven circularization, and RBP-driven circularization; and three types of circRNAs, including ciRNA, ecircRNA and EIciRNA.

Both circRNAs and long non-coding RNAs (lncRNAs) are derived from primary mRNA; however, circRNA splicing is unique from that of other lncRNAs as they are mainly produced via back-splicing, which involves ligating an upstream 3′ splice site with a downstream 5′ splice site, generating a closed-loop structure with a specific junction point [16]. CircRNAs are classified into three types according to their origins: exonic circle RNAs (ecircRNAs), circular intronic RNAs (ciRNAs), and exon–intron circular RNAs (EIciRNAs), with ecircRNAs accounting for roughly 80% of all the circRNAs that have been discovered [17] (Figure 1). Additionally, circRNAs undergo one of three commonly recognized back-splicing methods: lariat-driven, intron-pair-driven, and RNA-binding protein (RBP)-driven circularization (Figure 1). In lariat-driven circularization, the splicing donor covalently binds to the splicing acceptor through exon skipping, thus forming an exon-containing lariat and eventually forming ecircRNAs or EIciRNAs [13]. Furthermore, intron lariats with both a 7 nt GU-rich element and an 11 nt C-rich element can generate ciRNAs and resist debranching [18]. With intron-paring-driven circularization, complementary base pairs between introns bring the two exons together, and the spliceosome chops away the exons and introns to form ecircRNAs or EIciRNAs [19]. RBP-driven circularization involves the complementary base pairing of inverted repeats in the introns surrounding circRNA-forming exons [20]. The type of circRNA produced is mostly determined by the constituents in the three possible models and by the splicing pattern.

### 2.3. Functions

**Figure 2:**
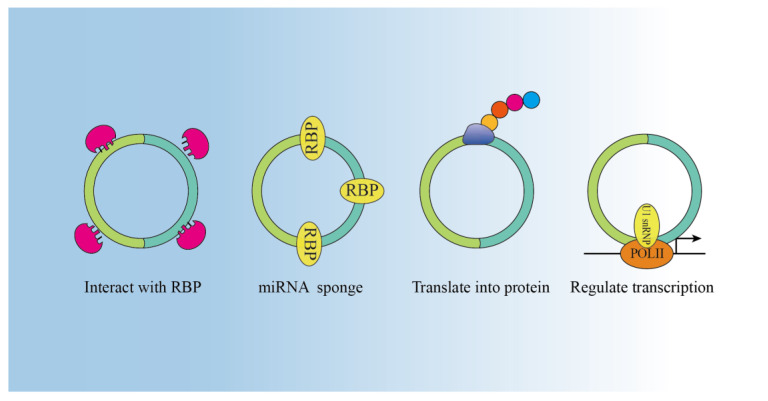
Functions of circRNAs: circRNAs function as miRNA sponges, thus regulating the expression of downstream genes. CircRNAs can bind with RBP. CircRNA can regulate transcription. Some circRNAs are able to be translated into protein.

With advances in circRNA research, the biological functions of circRNAs have been revealed. The main biological functions include acting as miRNA sponges, interacting with RBPs, encoding proteins, and regulating transcription (Figure 2).

#### 2.3.1. miRNAs Sponge

miRNAs are endogenous RNAs that can pair with the 3′ untranslated regions of mRNAs [21]. CircRNAs can interact with miRNAs and function as competing RNAs to eliminate the suppression of the miRNAs’ target gene of miRNA. CircRNAs play a crucial role in different cancers by acting as miRNA sponges. For instance, circPDSS1 promotes GC progression by acting as an miR-186-5p sponge, thus increasing NEK2 expression in gastric carcinoma [22]. CircMOT1 binds with miR-9, inhibiting the progression in hepatocellular carcinoma (HCC) [23]. Circ_0011946 can act as an miR-216a-5p sponge, promoting the expression of BCL2L2, whereas circ_0011946 knockdown constrains the tumorigenic phenotypes of OSCC cells [24]. These results collectively indicate that the sequestering of miRNAs is a common function for circRNAs. Many circRNAs contain many different types of miRNA response elements, regulating various biological processes. For example, the knockdown of circPVT1, first reported to bind with miR-125b, suppresses OSCC cell proliferation. Later, it was found to bind with miR-106a-5p, thereby influencing important biological processes [25].

#### 2.3.2. Interaction with RNA Binding Proteins (RBPs)

CircRNAs directly bind with proteins to regulate cell mechanisms, participating in the progression of physiology and pathology. RBPs, a general term for proteins that can recognize RNA, play a critical role in gene transcription and translation. The absence or dysfunction of RBPs may induce various diseases. Recent data have revealed that circRNAs may interact with and alter the function of RBPs. For example, human antigen (HuR) is upregulated in various human cancers, playing a crucial role in cancer progression [26]. Circ_0000745 is upregulated in OSCC cells and tissues and interacts with the HuR protein, thereby regulating the expression of CCND1 [27]. To summarize, circRNAs can combine with specific RBPs to facilitate or inhibit their functions.

#### 2.3.3. Encoded Proteins

CircRNAs were previously recognized as endogenous RNAs unable to be translated into protein. However, some circRNAs from the cytoplasm can be translated into proteins [28]. For instance, circZNF609 can act as a translation template for splicing-dependent and cap-independent translation. The product encoded by circZNF609 plays an important role in myogenesis [29]. CircSHPRH is ORF-driven by the internal ribosome entry sites (IRESs) and the protein it encodes is highly expressed in the brain [30]. In addition, Yi et al. found that circAXIN1 encodes a novel protein named AXIN1-295aa. AXIN1-295aa can activate the Wnt-signaling pathway, promoting GC tumorigenesis and progression by acting as an oncogenic protein [31]. However, less research has been conducted on the translational function of circRNAs in OSCC. CircRNAs have the potential to encode proteins; however, bioinformatics and other methods are required to determine their mechanisms.

#### 2.3.4. Transcription Regulation

CircRNAs regulate gene expression through various mechanisms, including transcription. CircRNAs can bind with U1 small nuclear ribonucleoprotein (U1 snRNP) to regulate the transcription of target genes [32]. For example, ci-ankrd52 is able to interact with the RNA polymerase complex to promote parental gene transcription [18].

## 3. Approaches for CircRNAs Studies in OSCC

Over the past decade, more studies concerning circRNAs have been conducted. In the past, RNA-sequencing (RNA-seq) technology was a popular tool for analyzing the differential expression of genes [33]. Additionally, microarrays, an efficient technology for circRNA profiling, were used to analyze OSCC tumor tissues and adjacent nontumor tissues to profile the significantly differentially expressed circRNAs in OSCC [34]. With the development of technology, high-throughput sequencing has become one of the main methods of sequencing circRNA [35]. Currently, several databases can be used for circRNA identification, quantification, annotation, and network identification to identify target and downstream signaling. Some examples of the databases are listed as follows (all accessed on 15 September 2023):

- circBase (http://www.circbase.org/) allows users to search for functional classes and processes [36];
- CircInteractome (https://circinteractome.nia.nih.gov/) contains the genome sequences and can predict the associated RBPs [37];
- LncRNADisease v3.0 (http://www.rnanut.net/lncrnadisease/) provides the circRNAs related to specific diseases [38].

A recent study introduced a method called MDGF-MCEC to predict circRNA disease associations based on a multi-view dual attention graph convolution network (GCN) with cooperative ensemble learning [39]. MDGF-MCEC requires certain assays to verify aberrantly expressed circRNAs. Real-time quantitative polymerasechain reaction (RT-qPCR) and Northern blotting can be employed to verify circRNA expression. Additionally, to investigate the relationships between circRNAs and OSCC, the overexpression/inhibition of circRNAs is the main method used to explore their functional implications. Some approaches can be used to overexpress or knockdown circRNAs, including: the CRISPR/Cas9-mediated circRNA knockout strategy [40]; using short interfering RNAs (siRNAs) to induce the circRNA cleavage strategy [41]; the CRISPR/Cas13-mediated circRNA knockdown strategy [42]; and the circRNA expression plasmid strategy [43]. Moreover, RNase R can be used to verify the circular structure of circRNAs. RNA pulls down assays, luciferase reporter assays, immunohistochemical analyses, and Western blotting have been widely applied in the research of circRNAs in OSCC. Overexpression vectors and siRNA are also commonly used to study the migration, invasion, and proliferation of OSCC cell lines. The above-mentioned methods are commonly used for the detection and verification of circRNAs in OSCC.

## 4. Research and Discovery of CircRNAs in OSCC

**Figure 3:**
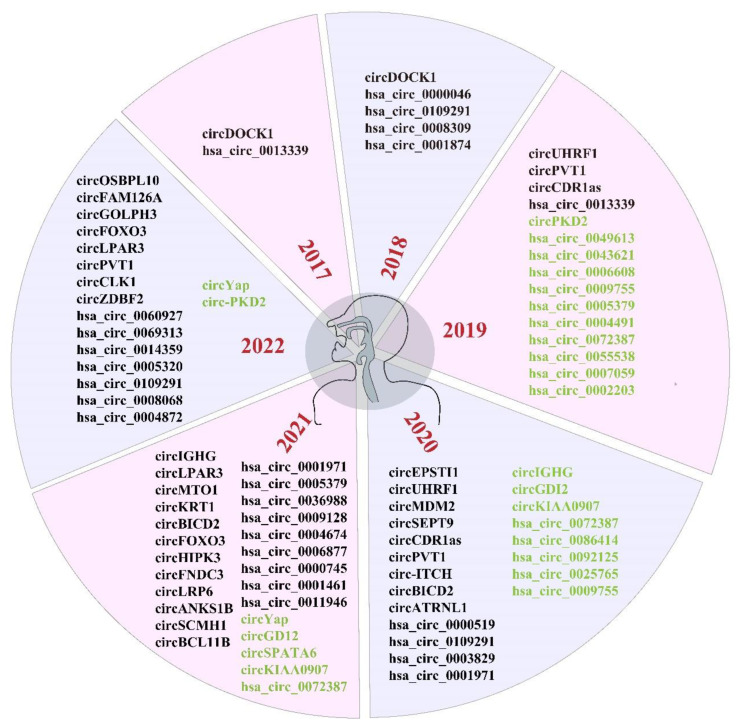
Research and discovery of circRNAs in oral squamous cell carcinoma (OSCC). The circRNAs labeled in green imply the upregulation of expression in OSCC.

Most circRNAs have been identified and characterized with the advancement of high-throughput sequencing and bioinformatic analyses. Since 2017, the number of publications on circRNAs in OSCC has increased annually. Approximately 120 differentially expressed circRNAs were identified from 2017 to 2022 (Figure 3), most of which are significantly overexpressed. Here, we summarized the mechanism of partial circRNAs in OSCC (Table 1).

### 4.1. Profiles of CircRNAs Expression in OSCC

Accumulating evidence demonstrates that most circRNAs are dysregulated in OSCC cells and tissues. According to Wang et al., 10,021 novel circRNA molecules were identified, of which eight are upregulated and eight are downregulated in OSCC patients [56]. Similarly, Deng et al. identified 213 differentially expressed circRNAs, of which 124 were upregulated and 89 were downregulated [57]. Using high-throughput sequencing, Wang et al. identified the expression profiles of circRNAs and confirmed that hsa_circ_009755 is significantly downregulated in OSCC, and is associated with T stage I–II in patients with OSCC [58]. Shaoet et al. found 122 differentially expressed circRNAs, among which 109 were upregulated and 13 were downregulated in OSCC. Additionally, research has reported 12 circRNAs, identified for the first time, as novel circRNAs [59]. These studies indicate that circRNAs participate in the progression of OSCC, making them potential therapeutic targets.

### 4.2. Distribution of CircRNAs in OSCC

**Figure 4:**
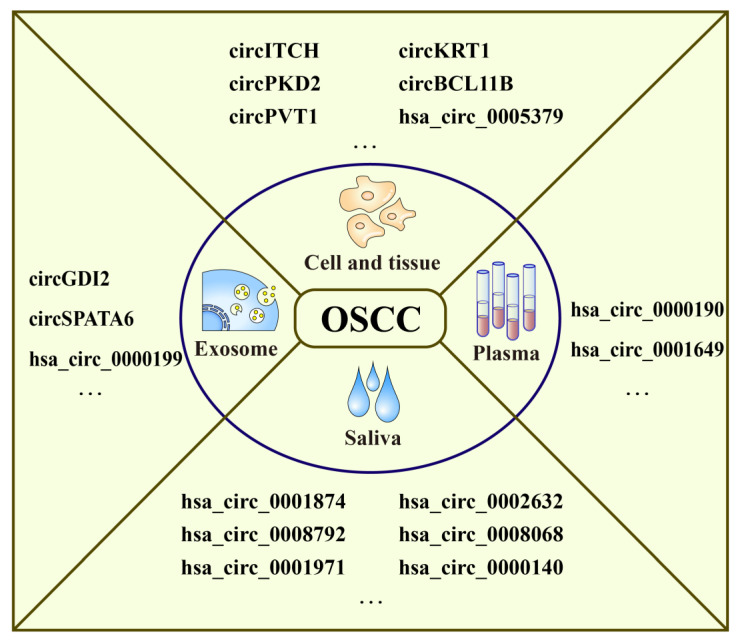
Distribution of circRNAs. CircRNAs are distributed in plasma, saliva, exosome, cells, and tissues. Some circRNAs related to OSCC are listed here.

CircRNAs are mainly located in the cell nucleus, but they have also been detected in the plasma, exosomes, saliva, cell, and tissue (Figure 4). Maass et al. predicted that circRNAs can be secreted into the plasma [60]. Vilades et al. discovered that hsa_circ_0001445 can stably exist in the plasma, and its expression is strongly correlated with atherogenic conditions [61]. Exosomes are nanoscale extracellular vesicles of endocytic origin [62], and exosomal circRNAs are a component of their contents [63]. In OSCC, exosomal circRNAs play important roles in the cell cycle and apoptosis and can hinder tumor cell growth [64]. In another study, the circRNA circ_0000199, which is highly expressed in exosomes, was associated with betel chewing, tumor size, lymphatic metastasis, and TNM stage in OSCC patients, and positively corrected with poor survival outcome [65]. Additionally, Zhang et al. proposed that upregulated exosomal circGDI2 weakens OSCC cell proliferation, migration, invasion, and glycolysis by competitively sponging miR-424-5p, regulating the expression of SCAI [66]. CircRNAs, which are stable and easily detectable in saliva, are promising biomarkers for OSCC diagnosis. Zhao et al. performed microarray screening, resulting in the identification of 12 upregulated and 20 downregulated circRNAs in saliva, among which hsa_circ_0001874, hsa_circ_0001971, and hsa_circ_0008068 were upregulated, whereas hsa_circ_0000140, hsa_circ_0002632, and hsa_circ_0008792 were downregulated. Salivary hsa_circ_0001874 was correlated with TNM stage and tumor grade, and hsa_circ_0001971 was correlated with TNM stage [67]. In a later study, Wang et al. found that hsa_circ_0001971 and hsa_circ_0001874 promoted cell proliferation by synergistically and collectively activating SHP2/PLK1 signals through sponging miR-186 and miR-296 [68]. Many circRNAs are aberrantly expressed in OSCC cells and tissues, and they are involved in the progression of OSCC.

## 5. CircRNA Pathways in OSCC

To date, most circRNAs have been reported to participate in the progression of OSCC by modulating various signaling pathways, such as the AKT/mTOR, Hippo, PD-1/PD-L1, and MAPK pathways. Here, we summarize the various pathways through which circRNAs are engaged in OSCC.

### 5.1. AKT/mTOR

The AKT/mTOR signaling pathway modulates various cellular processes, such as autophagy and the cell cycle, and regulates different hallmarks of cancer, including survival, invasion, migration and proliferation [69]. The key proteins involved in the regulation of this pathway are phosphoinositide 3-kinase (PI3K), AKT, and mTOR proteins [70]. Ferreira et al. discovered that AKT and mTOR proteins are abundant in OSCC tissues, indicating that the activation of the AKT/mTOR pathway is involved in the development of OSCC [71]. Since then, many circRNAs have been found to be involved in the progression of OSCC through the AKT/mTOR signaling pathway. The circRNA circCDR1as, which serves as a miR-671-5p sponge, activates phosphorylated AKT and ERK proteins and attenuates the phosphorylation of mTOR and reactive oxygen species (ROS) to enhance autophagy in OSCC [72]. In another study, hsa_circ_0007059 was shown to be upregulated in OSCC patients, downregulating the expressions of Bcl-2, MMP-9 and cyclin D1 but upregulating Bax, triggering the AKT/mTOR signaling pathway, resulting in the inhibition of cell proliferation, migration, and invasion, and the promotion of apoptosis [73]. To date, several AKT/mTOR inhibitors have been considered for the treatment of OSCC in preclinical and clinical studies, including natural and synthetic inhibitors [69]. In summary, most circRNAs participate in the progression of OSCC via the AKT/mTOR signaling pathway, suggesting that therapeutic agents could be developed for OSCC treatment by targeting these circRNAs.

### 5.2. Hippo Signaling Pathway

The Hippo signaling pathway, which is a conserved pathway, controls cell proliferation, tissue regeneration, organ size, and tumorigenesis through the downstream transcription coactivators YAP and TAZ [74]. In recent years, the Dbf2-related kinases LATS1 and LATS2 (LATS1/2), the main kinase components of this pathway, have attracted more research attention [75]. When the Hippo signaling pathway is switched off or inactive, YAP and TAZ remain unphosphorylated; therefore, they are translocated into the nucleus to bind the TEAD group of transcription factors, thereby regulating the transcription of genes required for cell proliferation and survival. Numerous pharmacological, mechanical, and physical stimuli phosphorylate MST1/2 and LATS1/2 upon activation of the Hippo signaling pathway, which subsequently impacts various physiological processes [76]. CircRNAs are involved in the development of malignant cancers via the Hippo signaling pathway, including GC and HCC. For example, circLARP4 behaves as an miR-424-5p sponge, suppressing cell proliferation and invasion in a LATS1-dependent manner [77]. Zhang found that YAP activates Fos Related Activator-1 (Fra-1), promoting cell growth and colony formation [78]. Additionally, Shriwas found that RRBP1, promotes cisplatin resistance in OSCC by regulating the Hippo signal pathway [79]. CircRNA_0000140 suppresses OSCC cell growth and metastasis by targeting miR-31 to regulate the expression of LATS2, thus inhibiting the Hippo signaling pathway [80]. To date, despite the extensive studies conducted on circRNAs, only a few circRNAs that regulate OSCC via the Hippo signaling pathway have been discovered.

### 5.3. PD-1/PD-L1 Signaling Pathway

The PD-1/PD-L1 signaling pathway has attracted considerable attention due to its effectiveness as an immune therapeutic target in various tumors [81]. Circ_001678 binds with miR-326 to upregulate the expression of ZEB to activate the PD-1/PD-L1 pathway, thus promoting CD8 T cell apoptosis and inducing cell immune escape in NSCLS [82]. Similarly, circRNAs can regulate PD-L1 expression thereby regulating tumor immune escape in OSCC. Yang et al. found a significantly higher level of circKRT1 in OSCC cell lines and tissues, suggesting that high circKRT1 expression could be a predictor of poor prognosis for OSCC patients. Moreover, circKRT1 drives tumor progression and immune evasion by sponging miR-495-3p to regulate PD-L1 expression [44]. To date, only a few circRNAs have been found to participate in the progression of OSCC via the PD-1/PD-L1 signaling pathway.

### 5.4. Other Signaling Pathways

Additional signaling pathways have been reported to be involved in the progression of OSCC. NF-κB is a widespread signaling pathway that plays an important regulatory role in inflammation and immune response [83]. Liang et al. found that circZDBF2 is upregulated in OSCC cell lines and regulates IL-8 transcription via NF-κB signaling pathway in OSCC [84]. Therefore, circRNAs play a crucial role in OSCC progression through various signaling pathways.

## 6. Oncogenic and Antioncogenic CircRNAs in OSCC

**Figure 5:**
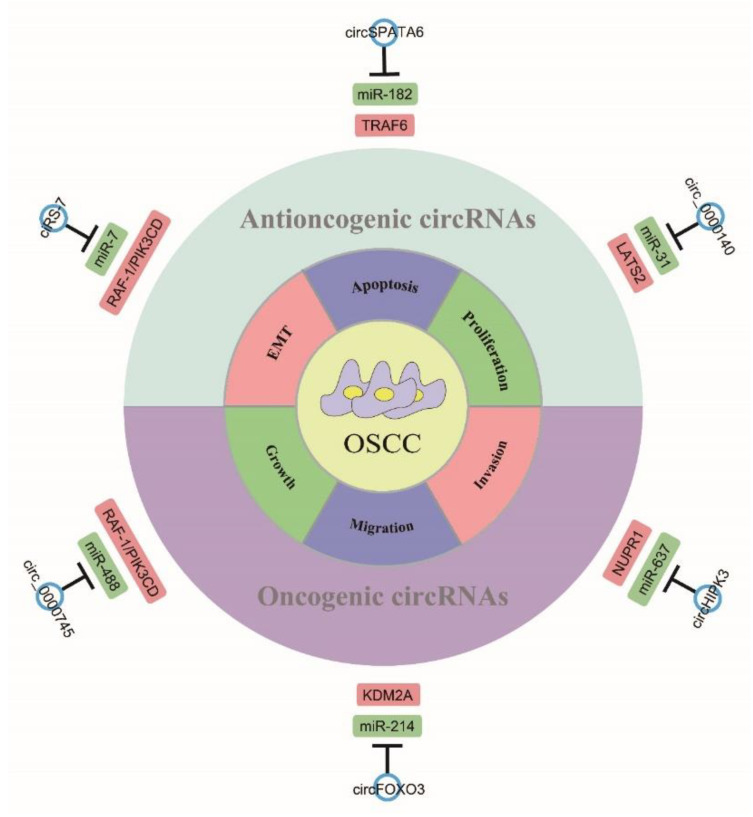
Oncogenic circRNAs and antioncogenic circRNAs. CircRNAs participate in OSCC progression via regulating cell apoptosis, proliferation, invasion, migration, growth, and EMT.

Generally, highly expressed circRNAs promote cell proliferation, migration, and invasion but inhibit apoptosis and cell cycle arrest in OSCC. In the published literature, the majority of circRNAs are reported to function as oncogenes, with a minority behaving as antioncogenic genes circRNAs in OSCC (Figure 5). Downregulated circRNAs typically function as tumor suppressors in OSCC, suppressing cell proliferation, migration, and invasion, and inducing cell cycle arrest and apoptosis.

### 6.1. Oncogenic CircRNAs in OSCC

#### 6.1.1. CircHIPK3

CircHIPK3 is generated from exon 2 of the HIPK gene [85]. CircHIPK3 can bind with multiple binding sites and plays a critical role in OSCC. Jiang et al. reported that circHIPK3 exerts oncogenic effects on OSCC progression by sponging miR-637 to further inhibit the downstream target NUPR1 [86]. NUPR1 is synthesized in the cytoplasm and transported to the nucleus to mediate the cell stress response and apoptosis. Wang et al. found that circHIPK3 binds with miR-124 thus promoting OSCC proliferation [87]. Additionally, circHIPK3 can regulate YAP1 by functioning similarly to miR-381-3p, contributing to OSCC growth and development [88].

#### 6.1.2. CircFOXO3

CircFOXO3 consists of a circularized second exon of the FOXO3 mRNA [89], and it is significantly upregulated in OSCC cells and tissues. Knocking down circFOXO3 prevents cell proliferation and substantially reduces OSCC cell invasiveness, suggesting that circFOXO3 plays an important role in the progression and invasion of OSCC. CircFOXO3 behaves as a miR-214 sponge thus upregulating the expression of KDM2A. CircFOXO3 can promote tumor growth and metastasis by decreasing miR-214 expression and increasing the expression of the miR-214 target KDM2A. Here, circFOXO3-miR214-KDM2A seems to play an important role in OSCC [90].

#### 6.1.3. Circ_0000745

The circRNA circ_0000745 was reported to be highly expressed in OSCC tissues and cell lines and its elevated expression was positively correlated with the poor overall survival of OSCC patients. has Additional studies have indicated that the downregulation of circ_0000745 inhibits OSCC development both in vitro and in vivo, including inhibiting colony formation, promoting cell apoptosis, and decreasing tumor volume, size and weight in mice. The application of bioinformatics tools and multiple assays confirmed that miR-488 is a target of circ_0000745, and the results of functional assays revealed that miR-488 inhibition reverses the inhibitory effects of circ_0000745 knockdown on OSCC development. CCND1 is the direct target of miR-488, and circ_0000745 competitively binds to miR-488 to regulate the expression of CCND1. Mechanistically, circ_0000745 acts as a sponge, abrogating the function of miR-103a-3p via direct binding, thus partly regulating the expression of CCND1 by interacting with the binding protein HuR to promote OSCC tumorigenesis and aggression [27].

### 6.2. Antioncogenic CircRNAs in OSCC

#### 6.2.1. CircSPATA6

CircSPATA6, derived from the exons of spermatogenesis-associated protein 6, inhibits OSCC progression through the sponging of miR-128. CircSPATA6 was previously confirmed to be frequently downregulated. Additionally, the overexpression of circSPATA6 suppresses migration, invasion, and cell cycle progression, and facilitates the apoptosis of OSCC cells by sponging miR-182. Bioinformatics and dual luciferase reporter assay results showed that TRAF6 serves as a target of miR-182. TRAF6 is implicated in the oncogenicity in OSCC, and the silencing of TRAF6 has been shown to abrogate miR-182-knockdown-mediated migration, invasion, progression, and apoptosis. Here, circSPATA6 was verified as a novel antioncogenic circRNA that regulates OSCC proliferation and apoptosis through the miR-182/TRAF6 regulatory network [64].

#### 6.2.2. CiRS-7

CiRS-7, one of the most studied circRNAs, is a miR-7 sponge [91]. CiRS-7 is involved in many different types of diseases, and act as antioncogenic circRNA through the MAPK/AKT signaling pathways. CiRS-7 is significantly upregulated in OSCC tissues and cells, and ciRS-7 significantly promotes OSCC cell proliferation, migration, and invasion both in vitro and in vivo. Moreover, bioinformatic predictions showed that ciRS-7 binds with miR-7, resulting in the attenuation of targets RAF-1 and PIK3D, which are core components of the MAPK/AKT signaling pathway. The results of a xenograft mice model showed that ciRS-7 overexpression promotes tumorigenicity by abrogating the miR-7-mediated suppressive effects on tumor growth. Studies further suggested that ciRS-7 weakens the effects of miR-7 concerning the downregulation of ERK1/2 and AKT phosphorylation in the MAPK/AKT pathway in vitro [92].

#### 6.2.3. Circ_0000140

In OSCC patients, circ_0000140, originating from exons 7 to 10 of the KIAA0907 gene, is downregulated and significantly correlated with higher lymph node metastases and a more advanced TNM stage at low expression. Additionally, miR-31 was found to be highly expressed in OSCC cell lines, and its expression was negatively associated with circ_0000140. The results of overexpression and knockdown experiments indicated that overexpressing circ_0000140 decreased the expression of miR-31 and increased the LATS2, P53, p-TAZ and p-YAP. Additionally, immunofluorescence analysis demonstrated that nuclear translocation of the YAP1 protein was attenuated in circ_0000140-overexpressing OSCC cells, suggesting the potential suppressive effect of circ_0000140 on the Hippo signaling pathway. Further results indicated that miR-31 directly targets LATS2, activating the Hippo signaling pathway in OSCC cells and inhibiting xenograft growth and lung metastasis in vivo. In summary, circ_0000140 inhibits tumor formation and lung metastasis via the epithelial mesenchymal transition (EMT) process by targeting miR-31 to repress the LATS2-mediated Hippo signaling [80].

## 7. Biological Functions of CircRNAs in OSCC

The dysregulation of circRNAs may be involved in the aberrant control of the processes in, and properties of, a number of cancer cell lines, including proliferation, apoptosis, metastasis, angiogenesis, and drug sensitivity.

### 7.1. Modulating the Tumor Microenvironment and Immune Escape

A major factor in the development and progression of cancer is the tumor microenvironment (TME). Immune cells are abundant in the TME and interact with circRNAs, thus participating in the proliferation, immune evasion, and metastasis of tumor cells [93,94,95]. Recently, several circRNAs associated with the TME were found to interact with immune cells, including T cells, B cells, natural killer cells (NKs) and macrophages [96]. Zou found that circCDR1as plays a specific role during cell infiltration in tumor tissues, especially in CD8 T cells, activated NK cells, M2 macrophages and endothelial cells [97]. Recently, circCDR1as was found to bind with miR-671-5p, enhancing OSCC cell survival, and circCDR1as levels were higher in OSCC tissues with high levels of HIF1α [72]. NK cells, the first line of defense for the host’s immunity, can secrete cytokines and chemokines that damage tumor cells [98]. CircARSP91 could upregulate UL16 binding protein 1 (ULBP1), thus enhancing the cytotoxicity of NK cells toward hepatocellular carcinoma [99]. CircRNAs can regulate the T-cell-mediated immune response; for example, hsa_circ_0005519 can regulate CD4 T cells, inducing the increase of IL-13 and IL-6 [100]. CircRNAs can transfer across to immune cells through exosomes or extracellular vesicles and act as tumor antigens to modulate immunity [101]. For example, the OSCC exosomes hsa_circ_0069313 were found to regulate Treg cells [52]. The TME is a hypoxic environment and HIF1α is a key component in the TME, playing an important role in infarction [102]. Tumor cells can escape from host T cells and, therefore the immune system, to prevent their clearance. PD-1/ PD-L1 is a crucial component in tumor immunosuppression, playing an important and diverse role in cell activation, tolerance, and immune-mediated tissue damage and tumorigenesis. CircRNAs can regulate the expression of PD-L1 to modulate immunity escape; hsa_circ_0069313 is positively associated with the expression of PD-L1. Additionally, the aberrantly high expression of hsa_circ_0069313 promotes the progression of OSCC by targeting Foxp3 by sponging miR-325-3p, thus decreasing the levels of Foxp3 and PD-L1 to enable immune escape.

### 7.2. Modulating Chemoresistance and Radiosensitivity

Due to the complex etiologies and clinical manifestations of OSCC, various chemotherapeutic agents are used in its clinical treatment, such as cisplatin (DPP) and 5-FU [103,104]. Mechanistically, cisplatin combines with genomic DNA or mitochondrial DNA to form DNA–platinum adducts after entering OSCC cells, arresting DNA replication, inhibiting cell mitosis, and inducing apoptosis [105]. Evidence shows that non-coding RNAs, including circRNAs, have the potential to function as therapeutic targets for alleviating OSCC resistance [106]. In recent years, circRNAs have been recognized as regulators of cancer chemoresistance and radioresistance. For instance, the upregulation of circANKS1B increases transforming growth factor β (TGF-β1) expression via sponging, thus resulting in cisplatin resistance. In addition, recent studies have shown the expression of circANKS1B binds with miR-515-5p, thus interrupting DPP [48]. Another study on OSCC showed that circSCMH1 is upregulated in DDP-resistant cell lines and its knockdown makes the cell line more sensitive to DDP [107]. Circ_0001971 is known to be highly expressed in OSCC cells and tissues, and knocking down the former inhibits the latter’s invasion, migration, infiltration, and increases its sensitivity to DPP [108]. In addition, circ_0109291 knockdown was found to decrease the expression of canonical drug-resistance-related gene ABCB1, improving the sensitivity of OSCC cells to DDP [109]. The aberrant expression of circATRNL1 can induce cell apoptosis and cell cycle arrest, and circATRNL1 can bind with miR-23a-3p, thus increasing the level of PTEN to promote the radiosensitivity of OSCC cells [110]. Additionally, N6-methyladenosine (m6A), a prevalent RNA modification in eukaryotes, has major impacts on therapeutic resistance. The role of m6A and circRNAs is concentrated on four aspects: the biogenesis, cytoplasmic export, degradation, and translation of circRNAs [111]. For example, the expression of circRNA-SORE is more stable when modulating m6A, and behaves similarly to miR-103a-2-5p and miR-660-3p, thus activating the Wnt/β-catenin pathway, and contributing to sorafenib tolerance [112,113].

### 7.3. Modulating EMT

EMT is characterized by a loss of epithelial cells and their transformation into mesenchymal cells, which have the ability to move freely [114]. CircRNAs have been reported to also be involved in regulating the progression of OSCC through EMT. For instance, circFAM126A levels markedly increased in OSCC cells and tissues, and were positively associated with the EMT phenotype. CircFAM126A induces EMT by targeting miR-186, thus promoting OSCC progression [47]. Furthermore, hsa_circ_0009128 is correlated with advanced TNM stage and lymph node metastasis in OSCC patients. Mechanistically, hsa_circ_0009128 can target the decreasing matrix metalloprotein 9 (MMP9), thus promoting EMT, proliferation, and migration in OSCC cells [115]. The overexpression of circIGHG upregulates IGF2BP3 by suppressing miR-142-5p and inducing EMT, favoring the invasion and metastasis of OSCC [116].

### 7.4. Modulating Angiogenesis

Angiogenesis is essential for the growth and development of tumors because it allows fast-growing malignant tissues to be continuously fed with nutrients and oxygen while being cleansed of metabolic waste [117]. Vascular endothelial growth factors (VEGFs) promote blood vessel development via vasculogenesis. and angiogenesis. They are some of the most important pro-angiogenic factors secreted by tumor cells, which provide the large amounts of glucose and oxygen needed for tumor cells to thrive [118]. CircRNAs can directly regulate the expression of VEGFA, thus stimulating angiogenesis. For example, hsa_circ_0001766, acting as a miR-877-3p sponge, increases the expression of VEGFA, promoting the growth of OSCC cells [59]. CircRNAs are regulated by hypoxia and involved in angiogenesis [119]. In glioma, the silencing of circZNF292 suppresses tube formation via the Wnt/β-catenin pathway [120]. Knocking down circ_0005320 can suppress angiogenesis via the miR-486-3p/miR-637 axis in OSCC [46]. In another study, a tube formation assay showed that circLPAR3 decreased the number of tube formations, suggesting that circLPAR3 promotes the angiogenesis of OSCC cells. MiR-513b-5p is the direct target of circLPAR3, and the overexpression miR-513b-5p or knockdown of circLPAR3 suppresses the expression of AKT1 and VEGFC in OSCC cells. CircLPAR3 knockdown led to more keratin pearls in tumor tissues and inhibited the expression of CD34, a canonical protein marker of micro-vessel density. Furthermore, VEGFC and p-AKT1 were inhibited in tumors where circLPAR3 was downregulated, suggesting that the knockdown of circLPAR3 inhibits angiogenesis and tumor growth [121].

### 7.5. Modulating Endoplasmic Reticulum

Endoplasmic reticulum (ER) stress is a type of stress response that can result in cell death, autophagy, and unfolded protein responses (UPR) [122]. A close functional relationship between ER stress and circRNAs exists; they can regulate each other to determine the fate of tumor cells [123]. At present, three UPR signaling pathways have been reported to be involved in carcinogenesis: IRE1α-XBP1, PERK-eIF2α, and ATF6α [124,125,126]. The C/EBP homologous protein (CHOP) is an important transcription factor involved in regulating apoptosis. Non-coding RNAs, including circRNAs and miRNAs, can directly or indirectly act on UPR pathway molecules to regulate intracellular homeostasis and affect carcinogenic processes [127]. Deng found that circRNA_101036 can act as a tumor suppressor in OSCC cells by inducing endoplasmic reticulum stress. The upregulation of circRNA_101036 can increase the levels of CHOP protein and ROS, inducing the unfolded protein response (UPR) and promoting apoptosis [128]. In another study, circCDR1as was highly expressed in OSCC cells, and the overexpression of circCDR1as increased eIF2α expression, inducing endoplasmic reticulum stress, thus increasing OSCC cell viability [72].

## 8. Clinical Application

### 8.1. Diagnostic and Prognostic Biomarkers

Patients diagnosed with OSCC at the advanced stage face increased challenges in treatment, worsening their outcomes [129]. Therefore, the identification of a biomarker is critical to enable the detection and diagnosis of OSCC at an earlier stage. Due to circRNAs been stable, specific, and easy to extract and detect, they have potential as novel biomarkers in different cancers. CircRNAs are dysregulated in various cancers, and the expression of circRNAs varies according to the TNM stage, metastasis, and tumor size. Zhang et al. found that hsa_circ_0003829 is significantly downregulated in OSCC and negatively correlated with lymph node metastasis status and TNM stage [130]. In another study, Zhao et al. identified differentially expressed circRNAs in saliva from three OSCC patients via circRNA microarray screening, reported that both hsa_circ_0001874 and hsa_circ_0001971 correlated with the TNM stage. Additionally, hsa_circ_0001874 correlated with the tumor grade. These results indicate that salivary hsa_circ_0001874 and hsa_circ_0001971 may serve as biomarkers for OSCC diagnosis [40]. Reports are increasingly showing that circRNAs may serve as necessary novel biomarkers and therapeutic targets for cancer treatment. However, the clinical feasibility of using circRNAs still needs to be verified using robust circRNA detection methods in a large cohort of OSCC patients.

### 8.2. Drug Development

Therapies based on circRNAs have attracted the attention of researchers due to their multiple advantages. Drug resistance is a major challenge in OSCC treatment. At present, there are several strategies for delivering drugs in vivo, including exosome and nanoparticle delivery. Exosomes are also currently being explored as delivery vehicles for circRNA-targeting agents and as circRNA expression vectors [131]. The advantage of a circRNA vector is that it can be received by many cell types to facilitate intercellular communication. Nanoparticles can carry therapeutic agents and deliver them to the disease-affected site [132]. However, the safety of nanoparticle delivery needs to be further explored. Importantly, siRNA nanoparticles have been approved for treatment and are currently under investigation in clinical trials [133]. In the future, more studies are needed to verify the availability of approaches to target circRNAs and to ensure that they can be used in clinical practices.

## 9. Conclusions

CircRNAs have been a prominent topic in tumor biology and therapy. Scientists have gradually elucidated the biogenesis, characteristics, and functions of circRNAs. Studies have demonstrated that circRNAs are aberrantly expressed in OSCC and involved in its progression. The majority of known circRNAs function as oncogenic circRNAs, and tumors begin to form when circRNAs become dysregulated. Currently, there are no particularly effective therapies for OSCC. In this review, we demonstrated that circRNAs are involved in many biological processes, including cell invasion, growth, apoptosis, and drug resistance. Due to the characteristics of circRNAs, they have considerable potential as biomarkers for OSCC diagnosis and prognosis, and as treatment targets or tools. Furthermore, circRNAs exist in the saliva, urine, and plasma, and thus can be easily detected. However, the exact mechanisms through which circRNAs are involved in the progression of OSCC remains unclear. In addition, studies on, and the application of, circRNAs in targeted therapy are limited. In fact, circRNAs remain largely unexplored. For example, we do not yet know how to deliver circRNAs to the target site or the precise mechanisms through which circRNAs are involved in the progression of cancer to enable their effective application in clinical therapies. CircRNAs generally regulate several pathways in OSCC cell invasion and proliferation, including Hippo, AKT/mTOR, and PD-1/PD-L1. Therefore, future comprehensive elaboration and exploration of the molecular mechanisms underlying the occurrence and progression of OSCC can identify novel circRNAs as effective therapeutic targets, thus improving the overall survival of patients with OSCC.

## Figures and Tables

**Table 1:** The mechanism of partial circRNAs in OSCC.

CircRNA	Expression	MiRNA	Downstream Target	Function	Reference
CircKRT1	up	miR-495-3p	PD-L1	CircKRT1 knockdown repressed OSCC cell growth, migration, invasion, the epithelial mesenchymal transition (EMT), and CD8 T cell apoptosis, but enhanced CD8 T cytotoxicity and percentage	[44]
circPVT1	up	miR-143-3p	SLC7A11/ p38/ERK MAPK	Silencing of circPVT1 or SLC7A11 suppressed proliferation, migration, and invasion and promoted apoptosis in OSCC cells	[45]
circ_0005320	up	miR-486-3p miR-637	JAK2/STAT3	Knockdown of circ_0005320 suppressed OSCC cell growth, migration, invasion, and induced cell apoptosis in vitro, as well as impeded tumor growth in vivo	[46]
circFAM126A	up	miR-186	RAB41	Knockdown of circFAM126A markedly suppressed the proliferation, migration, and invasion of OSCC cells in vitro and inhibited tumor growth and distant metastasis in vivo	[47]
circ_0011946	up	miR-216-5p	BCL2L2	Circ_0011946 knockdown impeded proliferation, migration, and invasion, but promoted apoptosis in OSCC cells	[24]
circANKS1B	up	miR-515-5p	TGF-β1	The knockdown of circANKS1B sensitized OSCC cells to cisplatin by suppressing cell viability and increasing cell apoptosis and caspase-3 activity	[48]
circ-PKD2	down	miR-204-3p	APC2	Overexpression of circ-PKD2 inhibited OSCC cell proliferation, migration, and invasion, induced apoptosis and cell cycle arrest	[49]
circ_0005379	down	miR-17-5p	ACOX1	After overexpressing circ_0005379, the activity and number of migrating and invading SCC15 cells, however, the apoptosis rate and expression level of E-cadherin protein were increased	[50]
circUHRF1	up	miR-526b-5p	c-Myc/TGF-β1/ESRP1	Promoted the proliferation, migration, invasion, and EMT	[51]
hsa_circ_0069313	up	miR-325-3p	Foxp3	Induced OSCC immunity escape	[52]
circMTO1	up	miR-320a	ATRX	CircMTO1 knockdown inhibited OSCC cell proliferation, migration, and invasion	[53]
circDHTKD1	up	miR-326	GAB1	CircDHTKD1 knockdown was shown to impede OSCC cell growth and metastasis but motivate apoptosis	[54]
circBCL11B	up	miR-579	LASP1	Silencing cirCBCL11B inhibited cell proliferation and migration, and also included cell apoptosis in OSCC cells	[55]

## Data Availability

No new data were created or analyzed in this study. Data sharing is not applicable to this article.
